# Supplementary material for: Genetic heterogeneity of the Spy1336/R28—Spy1337 virulence axis in Streptococcus pyogenes and effect on gene transcript levels and pathogenesis
Source: PLoS One. 2020 Mar 26;15(3):e0229064. doi: 10.1371/journal.pone.0229064 (PMC7098570; doi:10.1371/journal.pone.0229064)
Supplement: S1 Fig — Strains were grown at 37°C in THY medium. (PDF) [file pone.0229064.s001.pdf]

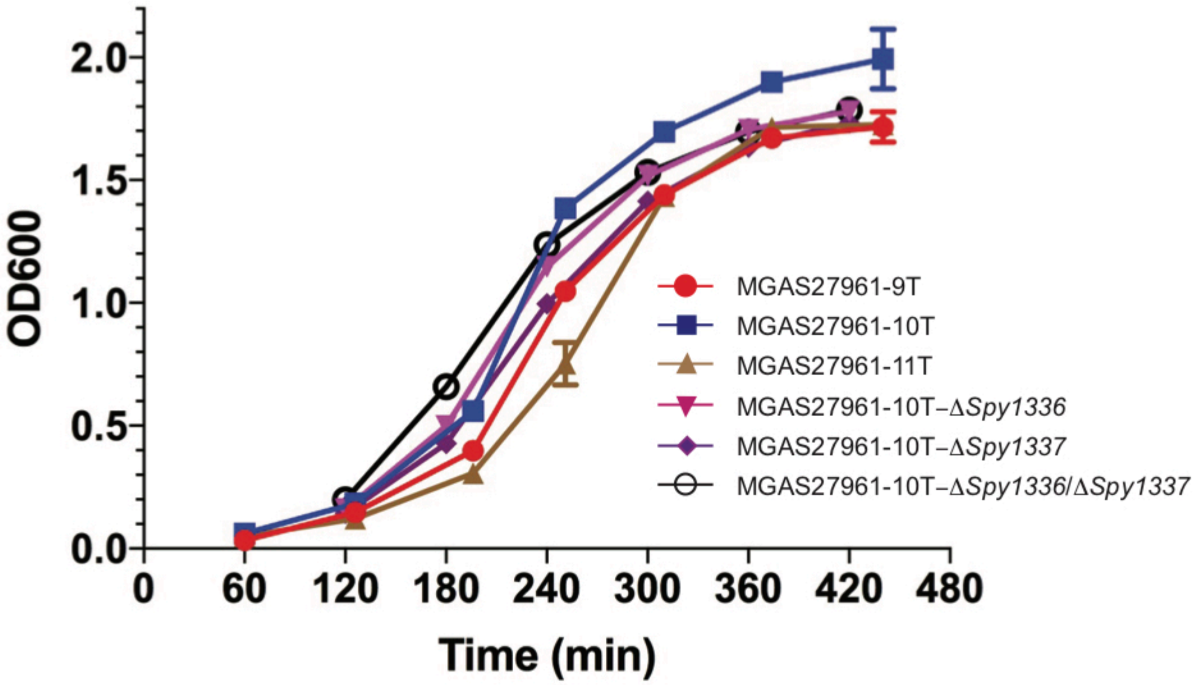

Supplementary Figure 1. Growth curves of isogenic mutants in THY. Strains were grown at 37°C in THY medium.
