# Supplementary material for: Genetic heterogeneity of the Spy1336/R28—Spy1337 virulence axis in Streptococcus pyogenes and effect on gene transcript levels and pathogenesis
Source: PLoS One. 2020 Mar 26;15(3):e0229064. doi: 10.1371/journal.pone.0229064 (PMC7098570; doi:10.1371/journal.pone.0229064)
Supplement: S2 Fig — (A) Total number of differentially expressed genes using a fold cutoff ≥1.5. Upregulated virulence genes comparing 9T vs 10T (B), 9T vs 11T (C), and 10T vs 11T (D). (PDF) [file pone.0229064.s002.pdf]

A. Number of differentially expressed genes

	10T vs 9T(1)		11T vs 9T(2)		11T vs 10T(3)		
	Number of genes	%(4)	Number of genes	%	Number of genes	%	Growth phase
Upregulated	18	1%	59	3.2%	8	0.4%	ME
Downregulated	13	0.7%	28	1.5%	7	0.4%	
Upregulated	102	5.6%	12	0.7%	8	0.4%	ES
Downregulated	154	8.4%	98	5.4%	5	0.3%	

(1) MGAS2761-10T compared to MGAS2761-9T
(2) MGAS2761-11T compared to MGAS2761-9T
(3) MGAS2761-11T compared to MGAS2761-10T
(4) Percentage of genes compared to the entire genome

B. Upregulated virulence genes (10T vs 9T)

	Growth	Spy	gene	Product	FC
1	ME	Spy0137	*nga*	NAD glycohydrolase	1.5
2	ME	Spy0138	*ifs*	immunity factor for SPN	1.6
3	ME	Spy0139	*slo*	streptolysin O	1.6
4	ME	Spy0540	*sagA*	streptolysin S precursor	1.6
5	ME	Spy1336	*Spy1336*	R28 protein	64.7
6	ME	Spy1337	*Spy1337*	transcriptional regulator, AraC family	4.3
7	ME	Spy1675	*sclA*	collagen-like surface protein A	2.4
8	ME	Spy1699	-	cell surface protein	1.6
9	ME	Spy1700	*scpA*	C5A peptidase precursor	1.7
10	ME	Spy1701	*enn*	enn protein	1.5
11	ME	Spy1702	*emm*	emm28 protein	1.7
1	ST	Spy0138	*ifs*	immunity factor for SPN	1.6
2	ST	Spy0139	*slo*	streptolysin O	1.5
3	ST	Spy1336	*Spy1336*	R28 protein	45.3
4	ST	Spy1337	*Spy1337*	transcriptional regulator, AraC family	7.6

C. Upregulated virulence genes (11T vs 9T)

	Growth	Spy	gene	Product	FC
1	ME	Spy0137	*nga*	NAD glycohydrolase	1.8
2	ME	Spy0138	*ifs*	immunity factor for SPN	2.0
3	ME	Spy0139	*slo*	streptolysin O	1.9
4	ME	Spy0329	*spyCEP*	lactocepin	2.9
5	ME	Spy0540	*sagA*	streptolysin S precursor	1.7
6	ME	Spy0541	*sagB*	streptolysin S biosynthesis protein	1.6
7	ME	Spy0542	*sagC*	streptolysin S biosynthesis protein	1.6
8	ME	Spy0543	*sagD*	streptolysin S biosynthesis protein	1.7
9	ME	Spy0544	*sagE*	streptolysin S putative self-immunity protein	1.8
10	ME	Spy0545	*sagF*	streptolysin S biosynthesis protein	1.7
11	ME	Spy0546	*sagG*	streptolysin S export ATP-binding protein	1.8
12	ME	Spy0547	*sagH*	streptolysin S export transmembrane protein	1.7
13	ME	Spy0548	*sagI*	streptolysin S export transmembrane protein	1.8
14	ME	Spy1336	*Spy1336*	R28 protein	115.0
15	ME	Spy1337	*Spy1337*	transcriptional regulator, AraC family	6.0
16	ME	Spy1675	*sclA*	collagen-like surface protein A	3.7
17	ME	Spy1699	-	cell surface protein	2.1
18	ME	Spy1700	*scpA*	C5A peptidase precursor	2.2
19	ME	Spy1701	*enn*	enn protein	1.9
20	ME	Spy1702	*emm*	emm28 protein	1.9
1	ST	Spy0137	*nga*	NAD glycohydrolase	1.9
2	ST	Spy0138	*ifs*	immunity factor for SPN	1.8
3	ST	Spy0139	*slo*	streptolysin O	1.7
4	ST	Spy1336	*Spy1336*	R28 protein	72.6
5	ST	Spy1337	*Spy1337*	transcriptional regulator, AraC family	9.1
6	ST	Spy1702	*emm*	emm28 protein	2.3
7	ST	Spy1716	*sof*	serum opacity factor	1.5

D. Upregulated virulence genes (11T vs 10T)

	Growth	Spy	gene	Product	FC
1	ME	Spy0329	*spyCEP*	lactocepin	1.5
2	ME	Spy1336	*Spy1336*	R28 protein	1.8
1	ST	Spy1336	*Spy1336*	R28 protein	1.6
2	ST	Spy1675	*sclA*	collagen-like surface protein A	1.7
3	ST	Spy1702	*emm*	emm28 protein	1.7
4	ST	Spy1621	*salA*	lantibiotic salivaricin A	1.8

Supplementary Figure 2. RNA-seq results comparing isogenic 9T, 10T, and 11T strains. (**A**) Total number of differentially expressed genes using a fold cutoff ≥1.5. Upregulated virulence genes comparing 9T vs 10T (**B**), 9T vs 11T (**C**), and 10T vs 11T (**D**).
