# Supplementary material for: Genetic heterogeneity of the Spy1336/R28—Spy1337 virulence axis in Streptococcus pyogenes and effect on gene transcript levels and pathogenesis
Source: PLoS One. 2020 Mar 26;15(3):e0229064. doi: 10.1371/journal.pone.0229064 (PMC7098570; doi:10.1371/journal.pone.0229064)
Supplement: S3 Fig — Number of TRR28 repeats in strains with HTSpy1336-7 containing 8Ts (A), 9Ts (B), 10Ts (C), and 11Ts (D). n = 490 strains. (PDF) [file pone.0229064.s003.pdf]

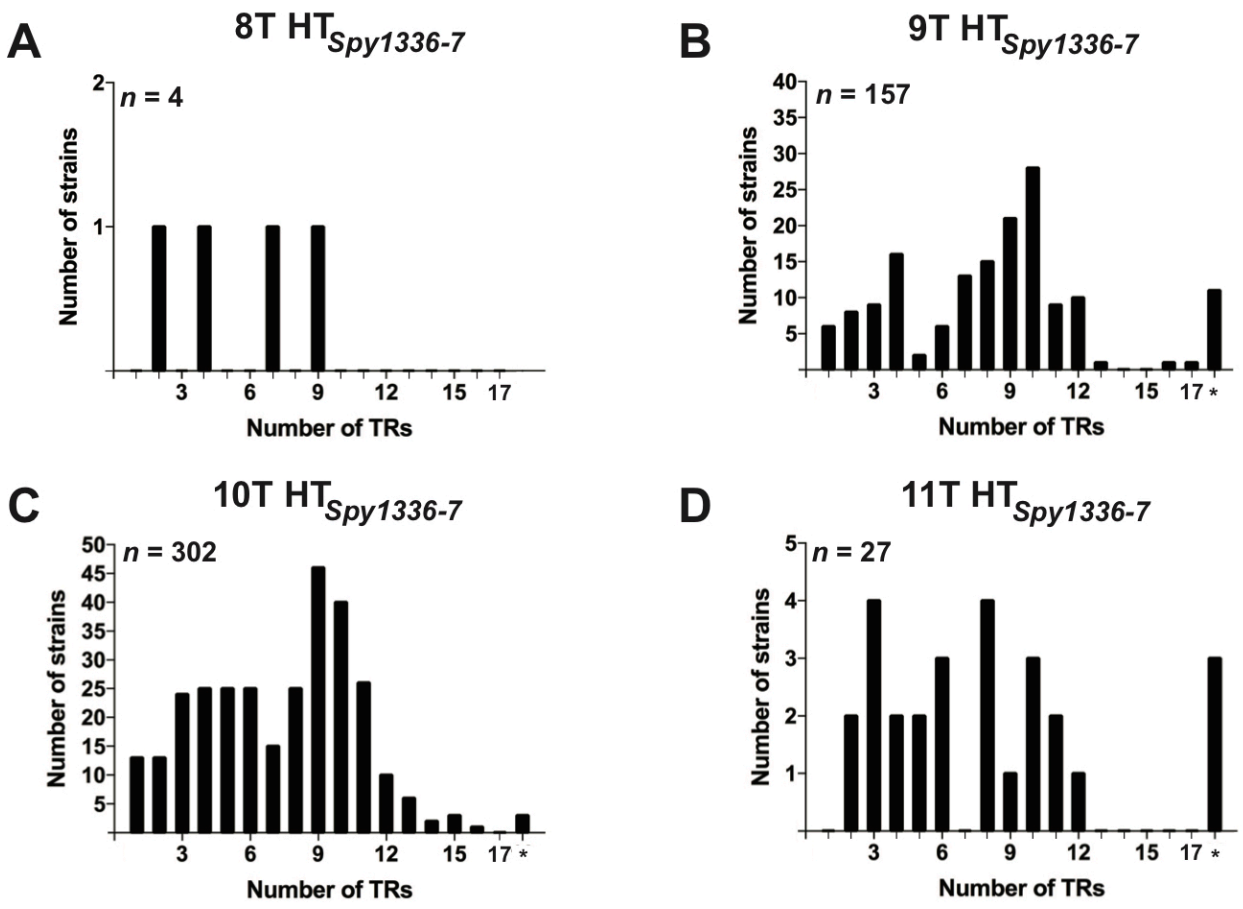


*, Not determined

The total number of strains is *n* = 490. Of the original 493 strains 2 have no RD2 and 1 has 13 Ts

**Supplementary Figure 3. Correlation between the number of $TR_{R28}$ repeats in Spy1336/R28 and the number of T nucleotides in the corresponding $HT_{\mathit{Spy1336-07}}$.** Number of $TR_{R28}$ repeats in strains with $HT_{\mathit{Spy1336-7}}$ containing 8Ts (**A**), 9Ts (**B**), 10Ts (**C**), and 11Ts (**D**). n=490 strains.
