# Supplementary material for: Genetic heterogeneity of the Spy1336/R28—Spy1337 virulence axis in Streptococcus pyogenes and effect on gene transcript levels and pathogenesis
Source: PLoS One. 2020 Mar 26;15(3):e0229064. doi: 10.1371/journal.pone.0229064 (PMC7098570; doi:10.1371/journal.pone.0229064)
Supplement: S1 Raw images — (PDF) [file pone.0229064.s004.pdf]

Figure 2C original image

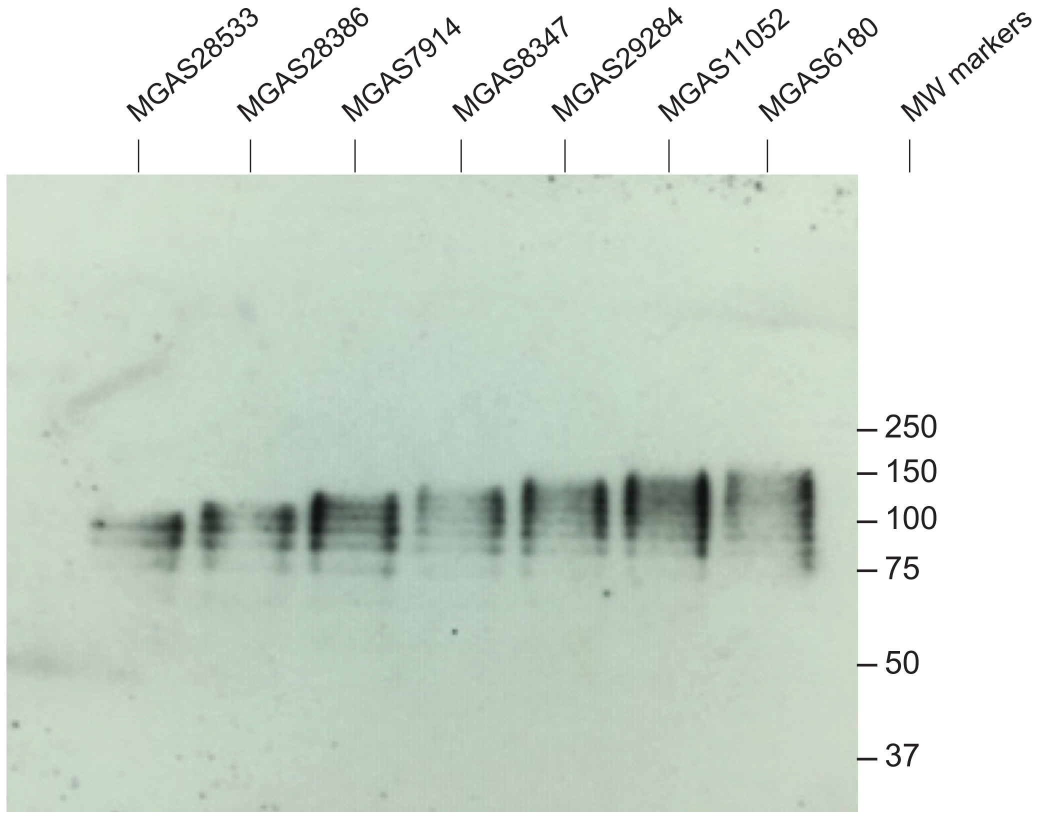

MW: Molecular weight

Original picture of a Kodak film placed on a transilluminator was taken using an iphone

Figure 6A original image

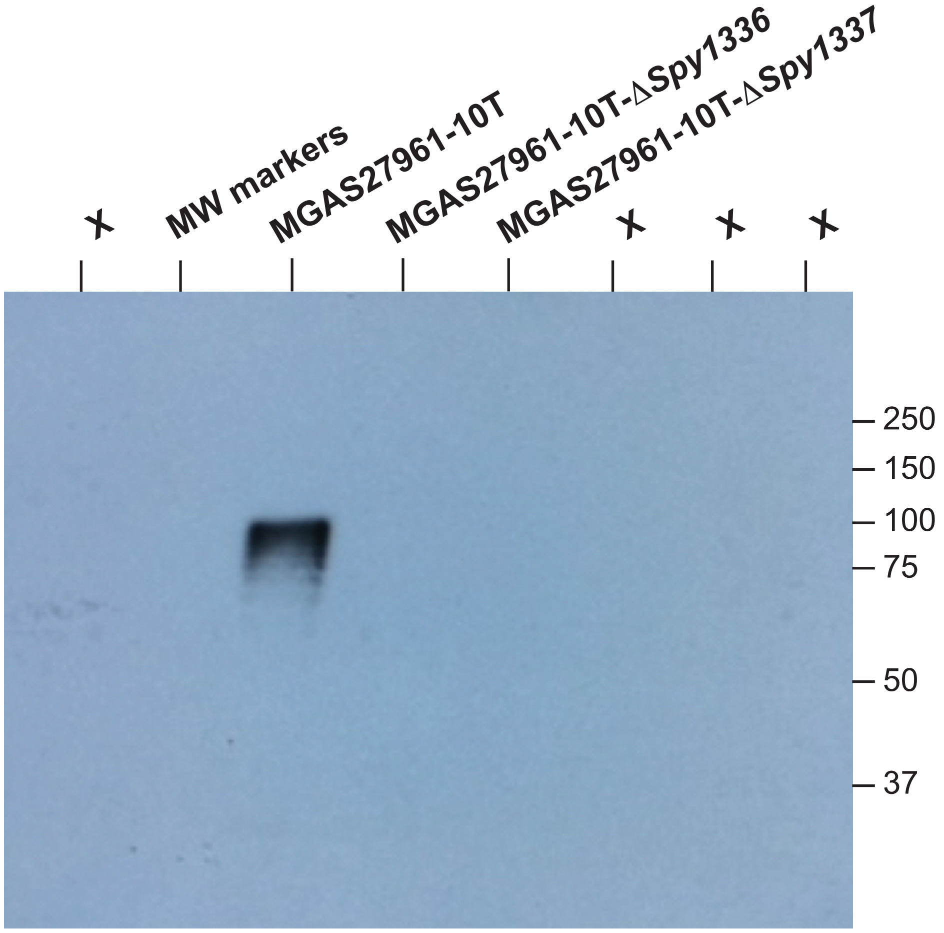

MW: Molecular weight

Original picture of a Kodak film placed on a transilluminator was taken using an iphone

Figure 6B original image

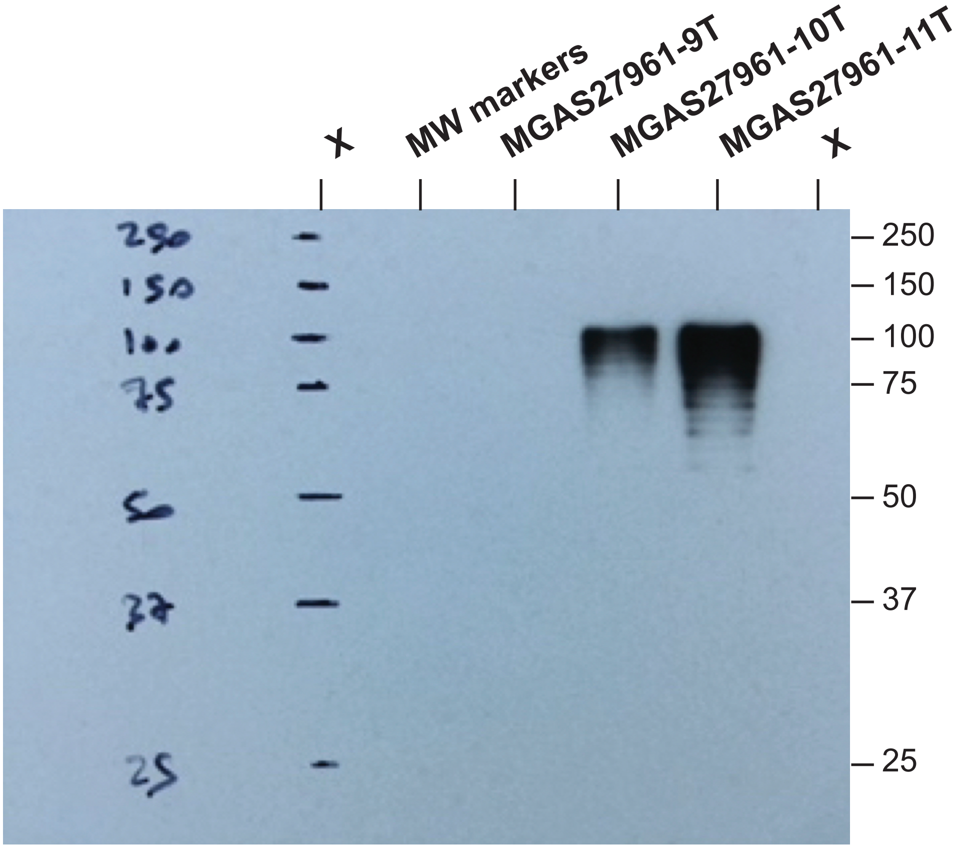

MW: Molecular weight

Original picture of a Kodak film placed on a transilluminator was taken using an iphone
