# Supplementary material for: Genetic heterogeneity of the Spy1336/R28—Spy1337 virulence axis in Streptococcus pyogenes and effect on gene transcript levels and pathogenesis
Source: PLoS One. 2020 Mar 26;15(3):e0229064. doi: 10.1371/journal.pone.0229064 (PMC7098570; doi:10.1371/journal.pone.0229064)
Supplement: S1 Table — (DOCX) [file pone.0229064.s005.docx]

**S1 Table. Number of T_R28_ repeats in 493 *emm28* invasive strains**

| **No.** | **MGAS  number** | **TR_R28_ number** | **Number of Ts in HT*_Spy1336-7_*** |
| --- | --- | --- | --- |
| 1 | **7867** | **1** | 10 |
| 2 | **8417** | **1** | 10 |
| 3 | **10820** | **1** | 10 |
| 4 | **11115** | **1** | 10 |
| 5 | **28035** | **1** | 10 |
| 6 | **28118** | **1** | 9 |
| 7 | **28201** | **1** | 10 |
| 8 | **28374** | **1** | 9 |
| 9 | **28463** | **1** | 10 |
| 10 | **28550** | **1** | 9 |
| 11 | **28776** | **1** | 10 |
| 12 | **28788** | **1** | 9 |
| 13 | **28935** | **1** | 10 |
| 14 | **29069** | **1** | 10 |
| 15 | **29080** | **1** | 9 |
| 16 | **29164** | **1** | 10 |
| 17 | **29431** | **1** | 10 |
| 18 | **29569** | **1** | 9 |
| 19 | **29587** | **1** | 10 |
| 20 | **11147** | **2** | 9 |
| 21 | **11151** | **2** | 9 |
| 22 | **12231** | **2** | 10 |
| 23 | **27846** | **2** | 10 |
| 24 | **28008** | **2** | 10 |
| 25 | **28323** | **2** | 10 |
| 26 | **28424** | **2** | 8 |
| 27 | **28484** | **2** | 10 |
| 28 | **28530** | **2** | 10 |
| 29 | **28633** | **2** | 10 |
| 30 | **28648** | **2** | 10 |
| 31 | **28764** | **2** | 10 |
| 32 | **28811** | **2** | 9 |
| 33 | **28842** | **2** | 10 |
| 34 | **29073** | **2** | 11 |
| 35 | **29074** | **2** | 9 |
| 36 | **29117** | **2** | 9 |
| 37 | **29253** | **2** | 9 |
| 38 | **29268** | **2** | 10 |
| 39 | **29276** | **2** | 10 |
| 40 | **29321** | **2** | 10 |
| 41 | **29372** | **2** | 9 |
| 42 | **29412** | **2** | 9 |
| 43 | **29611** | **2** | 11 |
| 44 | **7912** | **3** | 10 |
| 45 | **7927** | **3** | 10 |
| 46 | **7941** | **3** | 10 |
| 47 | **10757** | **3** | 10 |
| 48 | **10771** | **3** | 10 |
| 49 | **12254** | **3** | 10 |
| 50 | **28058** | **3** | 9 |
| 51 | **28059** | **3** | 10 |
| 52 | **28225** | **3** | 9 |
| 53 | **28232** | **3** | 11 |
| 54 | **28249** | **3** | 11 |
| 55 | **28273** | **3** | 11 |
| 56 | **28296** | **3** | 10 |
| 57 | **28305** | **3** | 10 |
| 58 | **28307** | **3** | 9 |
| 59 | **28331** | **3** | 10 |
| 60 | **28334** | **3** | 10 |
| 61 | **28356** | **3** | 9 |
| 62 | **28362** | **3** | 10 |
| 63 | **28425** | **3** | 10 |
| 64 | **28430** | **3** | 10 |
| 65 | **28443** | **3** | 9 |
| 66 | **28562** | **3** | 10 |
| 67 | **28566** | **3** | 9 |
| 68 | **28718** | **3** | 10 |
| 69 | **29109** | **3** | 9 |
| 70 | **29299** | **3** | 10 |
| 71 | **29338** | **3** | 10 |
| 72 | **29394** | **3** | 10 |
| 73 | **29476** | **3** | 10 |
| 74 | **29501** | **3** | 10 |
| 75 | **29508** | **3** | 11 |
| 76 | **29553** | **3** | 10 |
| 77 | **29570** | **3** | 9 |
| 78 | **29608** | **3** | 9 |
| 79 | **29617** | **3** | 10 |
| 80 | **7872** | **4** | 9 |
| 81 | **7886** | **4** | 10 |
| 82 | **7960** | **4** | 9 |
| 83 | **7995** | **4** | 10 |
| 84 | **8375** | **4** | 10 |
| 85 | **8447** | **4** | 9 |
| 86 | **11140** | **4** | 10 |
| 87 | **11149** | **4** | 10 |
| 88 | **27951** | **4** | 10 |
| 89 | **27970** | **4** | 9 |
| 90 | **27979** | **4** | 10 |
| 91 | **27983** | **4** | 11 |
| 92 | **28207** | **4** | 9 |
| 93 | **28271** | **4** | 10 |
| 94 | **28341** | **4** | 10 |
| 95 | **28367** | **4** | 10 |
| 96 | **28418** | **4** | 10 |
| 97 | **28440** | **4** | 10 |
| 98 | **28441** | **4** | 9 |
| 99 | **28446** | **4** | 10 |
| 100 | **28447** | **4** | 9 |
| 101 | **28481** | **4** | 9 |
| 102 | **28576** | **4** | 10 |
| 103 | **28650** | **4** | 10 |
| 104 | **28669** | **4** | 9 |
| 105 | **28687** | **4** | 10 |
| 106 | **28708** | **4** | 9 |
| 107 | **28724** | **4** | 10 |
| 108 | **28772** | **4** | 10 |
| 109 | **28784** | **4** | 10 |
| 110 | **28785** | **4** | 10 |
| 111 | **28822** | **4** | 10 |
| 112 | **28826** | **4** | 9 |
| 113 | **28880** | **4** | 10 |
| 114 | **28897** | **4** | 9 |
| 115 | **29050** | **4** | 10 |
| 116 | **29108** | **4** | 10 |
| 117 | **29138** | **4** | 9 |
| 118 | **29170** | **4** | 9 |
| 119 | **29184** | **4** | 10 |
| 120 | **29212** | **4** | 9 |
| 121 | **29342** | **4** | 10 |
| 122 | **29351** | **4** | 11 |
| 123 | **29384** | **4** | 9 |
| 124 | **29464** | **4** | 9 |
| 125 | **10819** | **5** | 10 |
| 126 | **10824** | **5** | 10 |
| 127 | **10826** | **5** | 9 |
| 128 | **28020** | **5** | 10 |
| 129 | **28029** | **5** | 10 |
| 130 | **28121** | **5** | 10 |
| 131 | **28127** | **5** | 9 |
| 132 | **28185** | **5** | 10 |
| 133 | **28402** | **5** | 10 |
| 134 | **28415** | **5** | 10 |
| 135 | **28461** | **5** | 10 |
| 136 | **28479** | **5** | 10 |
| 137 | **28543** | **5** | 10 |
| 138 | **28596** | **5** | 10 |
| 139 | **28622** | **5** | 10 |
| 140 | **28722** | **5** | 11 |
| 141 | **28751** | **5** | 10 |
| 142 | **28752** | **5** | 10 |
| 143 | **28804** | **5** | 10 |
| 144 | **28805** | **5** | 10 |
| 145 | **28942** | **5** | 10 |
| 146 | **29159** | **5** | 10 |
| 147 | **29198** | **5** | 10 |
| 148 | **29209** | **5** | 11 |
| 149 | **29305** | **5** | 10 |
| 150 | **29371** | **5** | 10 |
| 151 | **29423** | **5** | 10 |
| 152 | **29485** | **5** | 10 |
| 153 | **29537** | **5** | 10 |
| 154 | **7893** | **6** | 10 |
| 155 | **7946** | **6** | 10 |
| 156 | **10817** | **6** | 10 |
| 157 | **10827** | **6** | 10 |
| 158 | **27865** | **6** | 9 |
| 159 | **28047** | **6** | 10 |
| 160 | **28085** | **6** | 10 |
| 161 | **28142** | **6** | 10 |
| 162 | **28204** | **6** | 9 |
| 163 | **28278** | **6** | 10 |
| 164 | **28338** | **6** | 10 |
| 165 | **28347** | **6** | 10 |
| 166 | **28455** | **6** | 10 |
| 167 | **28462** | **6** | 10 |
| 168 | **28553** | **6** | 11 |
| 169 | **28567** | **6** | 10 |
| 170 | **28645** | **6** | 11 |
| 171 | **28647** | **6** | 10 |
| 172 | **28688** | **6** | 10 |
| 173 | **28710** | **6** | 10 |
| 174 | **28840** | **6** | 10 |
| 175 | **28883** | **6** | 10 |
| 176 | **29075** | **6** | 10 |
| 177 | **29133** | **6** | 10 |
| 178 | **29140** | **6** | 9 |
| 179 | **29243** | **6** | 10 |
| 180 | **29258** | **6** | 9 |
| 181 | **29293** | **6** | 10 |
| 182 | **29323** | **6** | 10 |
| 183 | **29474** | **6** | 9 |
| 184 | **29491** | **6** | 11 |
| 185 | **29502** | **6** | 9 |
| 186 | **29546** | **6** | 10 |
| 187 | **29613** | **6** | 10 |
| 188 | **7869** | **7** | 10 |
| 189 | **7976** | **7** | 10 |
| 190 | **7982** | **7** | 10 |
| 191 | **8358** | **7** | 9 |
| 192 | **28106** | **7** | 10 |
| 193 | **28208** | **7** | 9 |
| 194 | **28230** | **7** | 10 |
| 195 | **28360** | **7** | 9 |
| 196 | **28411** | **7** | 10 |
| 197 | **28426** | **7** | 9 |
| 198 | **28504** | **7** | 9 |
| 199 | **28508** | **7** | 9 |
| 200 | **28533** | **7** | 10 |
| 201 | **28549** | **7** | 9 |
| 202 | **28582** | **7** | 10 |
| 203 | **28589** | **7** | 10 |
| 204 | **28781** | **7** | 10 |
| 205 | **28787** | **7** | 10 |
| 206 | **28809** | **7** | 10 |
| 207 | **28845** | **7** | 9 |
| 208 | **28851** | **7** | 9 |
| 209 | **28872** | **7** | 8 |
| 210 | **28904** | **7** | 10 |
| 211 | **29051** | **7** | 10 |
| 212 | **29093** | **7** | 9 |
| 213 | **29180** | **7** | 9 |
| 214 | **29181** | **7** | 10 |
| 215 | **29324** | **7** | 9 |
| 216 | **29510** | **7** | 9 |
| 217 | **7956** | **8** | 10 |
| 218 | **8345** | **8** | 10 |
| 219 | **12247** | **8** | 9 |
| 220 | **27937** | **8** | 10 |
| 221 | **27956** | **8** | 10 |
| 222 | **27990** | **8** | 10 |
| 223 | **28004** | **8** | 9 |
| 224 | **28048** | **8** | 9 |
| 225 | **28082** | **8** | 9 |
| 226 | **28164** | **8** | 11 |
| 227 | **28209** | **8** | 10 |
| 228 | **28295** | **8** | 10 |
| 229 | **28321** | **8** | 10 |
| 230 | **28400** | **8** | 10 |
| 231 | **28585** | **8** | 9 |
| 232 | **28609** | **8** | 10 |
| 233 | **28612** | **8** | 10 |
| 234 | **28626** | **8** | 11 |
| 235 | **28801** | **8** | 9 |
| 236 | **28836** | **8** | 9 |
| 237 | **28869** | **8** | 9 |
| 238 | **28886** | **8** | 10 |
| 239 | **28900** | **8** | 9 |
| 240 | **28921** | **8** | 10 |
| 241 | **28924** | **8** | 10 |
| 242 | **29076** | **8** | 9 |
| 243 | **29095** | **8** | 10 |
| 244 | **29105** | **8** | 10 |
| 245 | **29188** | **8** | 9 |
| 246 | **29263** | **8** | 10 |
| 247 | **29300** | **8** | 9 |
| 248 | **29360** | **8** | 10 |
| 249 | **29381** | **8** | 9 |
| 250 | **29402** | **8** | 10 |
| 251 | **29416** | **8** | 10 |
| 252 | **29488** | **8** | 10 |
| 253 | **29504** | **8** | 10 |
| 254 | **29507** | **8** | 10 |
| 255 | **29534** | **8** | 9 |
| 256 | **29588** | **8** | 10 |
| 257 | **29594** | **8** | 11 |
| 258 | **29620** | **8** | 10 |
| 259 | **7890** | **9** | 10 |
| 260 | **7980** | **9** | 10 |
| 261 | **7994** | **9** | 10 |
| 262 | **8007** | **9** | 10 |
| 263 | **8009** | **9** | 9 |
| 264 | **10807** | **9** | 9 |
| 265 | **12250** | **9** | 9 |
| 266 | **27767** | **9** | 10 |
| 267 | **27787** | **9** | 10 |
| 268 | **27946** | **9** | 10 |
| 269 | **27967** | **9** | 10 |
| 270 | **27981** | **9** | 9 |
| 271 | **27993** | **9** | 9 |
| 272 | **28007** | **9** | 9 |
| 273 | **28022** | **9** | 10 |
| 274 | **28032** | **9** | 10 |
| 275 | **28046** | **9** | 10 |
| 276 | **28049** | **9** | 8 |
| 277 | **28074** | **9** | 10 |
| 278 | **28081** | **9** | 9 |
| 279 | **28086** | **9** | 10 |
| 280 | **28115** | **9** | 10 |
| 281 | **28138** | **9** | 9 |
| 282 | **28172** | **9** | 9 |
| 283 | **28241** | **9** | 9 |
| 284 | **28248** | **9** | 10 |
| 285 | **28265** | **9** | 10 |
| 286 | **28268** | **9** | 10 |
| 287 | **28287** | **9** | 10 |
| 288 | **28330** | **9** | 10 |
| 289 | **28386** | **9** | 11 |
| 290 | **28467** | **9** | 10 |
| 291 | **28471** | **9** | 10 |
| 292 | **28480** | **9** | 10 |
| 293 | **28482** | **9** | 10 |
| 294 | **28505** | **9** | 10 |
| 295 | **28510** | **9** | 10 |
| 296 | **28512** | **9** | 11 |
| 297 | **28513** | **9** | 10 |
| 298 | **28523** | **9** | 10 |
| 299 | **28630** | **9** | 10 |
| 300 | **28662** | **9** | 9 |
| 301 | **28707** | **9** | 10 |
| 302 | **28731** | **9** | 9 |
| 303 | **28744** | **9** | 10 |
| 304 | **28748** | **9** | 9 |
| 305 | **28798** | **9** | 10 |
| 306 | **28906** | **9** | 9 |
| 307 | **29161** | **9** | 10 |
| 308 | **29162** | **9** | 10 |
| 309 | **29214** | **9** | 9 |
| 310 | **29240** | **9** | 9 |
| 311 | **29254** | **9** | 10 |
| 312 | **29326** | **9** | 9 |
| 313 | **29331** | **9** | 10 |
| 314 | **29377** | **9** | 9 |
| 315 | **29379** | **9** | 10 |
| 316 | **29382** | **9** | 9 |
| 317 | **29387** | **9** | 10 |
| 318 | **29409** | **9** | 10 |
| 319 | **29426** | **9** | 10 |
| 320 | **29429** | **9** | 10 |
| 321 | **29444** | **9** | 10 |
| 322 | **29511** | **9** | 9 |
| 323 | **29542** | **9** | 9 |
| 324 | **29564** | **9** | 9 |
| 325 | **29583** | **9** | 10 |
| 326 | **7888** | **10** | 10 |
| 327 | **7891** | **10** | 9 |
| 328 | **7914** | **10** | 10 |
| 329 | **7921** | **10** | 10 |
| 330 | **7935** | **10** | 10 |
| 331 | **7959** | **10** | 10 |
| 332 | **7973** | **10** | 10 |
| 333 | **8012** | **10** | 10 |
| 334 | **8347** | **10** | 11 |
| 335 | **8365** | **10** | 10 |
| 336 | **8396** | **10** | 10 |
| 337 | **10786** | **10** | 10 |
| 338 | **10793** | **10** | 9 |
| 339 | **10799** | **10** | 10 |
| 340 | **10812** | **10** | 9 |
| 341 | **11103** | **10** | 9 |
| 342 | **11107** | **10** | 10 |
| 343 | **27961** | **10** | 9 |
| 344 | **27962** | **10** | 10 |
| 345 | **28031** | **10** | 9 |
| 346 | **28065** | **10** | 10 |
| 347 | **28107** | **10** | 10 |
| 348 | **28108** | **10** | 9 |
| 349 | **28117** | **10** | 9 |
| 350 | **28191** | **10** | 10 |
| 351 | **28217** | **10** | 10 |
| 352 | **28254** | **10** | 9 |
| 353 | **28261** | **10** | 10 |
| 354 | **28309** | **10** | 9 |
| 355 | **28315** | **10** | 9 |
| 356 | **28392** | **10** | 10 |
| 357 | **28397** | **10** | 9 |
| 358 | **28429** | **10** | 9 |
| 359 | **28473** | **10** | 10 |
| 360 | **28477** | **10** | 10 |
| 361 | **28501** | **10** | 10 |
| 362 | **28536** | **10** | 9 |
| 363 | **28640** | **10** | 10 |
| 364 | **28653** | **10** | 10 |
| 365 | **28654** | **10** | 9 |
| 366 | **28670** | **10** | 10 |
| 367 | **28686** | **10** | 9 |
| 368 | **28715** | **10** | 10 |
| 369 | **28728** | **10** | 10 |
| 370 | **28739** | **10** | 9 |
| 371 | **28746** | **10** | 9 |
| 372 | **28747** | **10** | 10 |
| 373 | **28825** | **10** | 10 |
| 374 | **28831** | **10** | 10 |
| 375 | **28864** | **10** | 9 |
| 376 | **28879** | **10** | 9 |
| 377 | **28898** | **10** | 9 |
| 378 | **28925** | **10** | 11 |
| 379 | **28949** | **10** | 9 |
| 380 | **29041** | **10** | 10 |
| 381 | **29045** | **10** | 10 |
| 382 | **29054** | **10** | 10 |
| 383 | **29068** | **10** | 10 |
| 384 | **29086** | **10** | 10 |
| 385 | **29124** | **10** | 10 |
| 386 | **29125** | **10** | 10 |
| 387 | **29201** | **10** | 10 |
| 388 | **29216** | **10** | 10 |
| 389 | **29221** | **10** | 9 |
| 390 | **29233** | **10** | 9 |
| 391 | **29238** | **10** | 10 |
| 392 | **29281** | **10** | 10 |
| 393 | **29310** | **10** | 10 |
| 394 | **29325** | **10** | 9 |
| 395 | **29330** | **10** | 10 |
| 396 | **29347** | **10** | 11 |
| 397 | **29404** | **10** | 9 |
| 398 | **29517** | **10** | 10 |
| 399 | **29559** | **10** | 9 |
| 400 | **29573** | **10** | 9 |
| 401 | **7918** | **11** | 10 |
| 402 | **7922** | **11** | 9 |
| 403 | **7987** | **11** | 10 |
| 404 | **8350** | **11** | 10 |
| 405 | **8374** | **11** | 10 |
| 406 | **10764** | **11** | 10 |
| 407 | **10802** | **11** | 10 |
| 408 | **12226** | **11** | 10 |
| 409 | **12248** | **11** | 9 |
| 410 | **27895** | **11** | 10 |
| 411 | **28041** | **11** | 9 |
| 412 | **28255** | **11** | 9 |
| 413 | **28317** | **11** | 10 |
| 414 | **28449** | **11** | 10 |
| 415 | **28557** | **11** | 10 |
| 416 | **28657** | **11** | 11 |
| 417 | **28661** | **11** | 9 |
| 418 | **28673** | **11** | 10 |
| 419 | **28699** | **11** | 10 |
| 420 | **28716** | **11** | 9 |
| 421 | **28760** | **11** | 11 |
| 422 | **28907** | **11** | 10 |
| 423 | **28932** | **11** | 10 |
| 424 | **29043** | **11** | 9 |
| 425 | **29061** | **11** | 10 |
| 426 | **29094** | **11** | 10 |
| 427 | **29143** | **11** | 9 |
| 428 | **29147** | **11** | 10 |
| 429 | **29230** | **11** | 10 |
| 430 | **29284** | **11** | 10 |
| 431 | **29286** | **11** | 10 |
| 432 | **29292** | **11** | 10 |
| 433 | **29314** | **11** | 10 |
| 434 | **29447** | **11** | 13 |
| 435 | **29494** | **11** | 9 |
| 436 | **29528** | **11** | 10 |
| 437 | **29599** | **11** | 10 |
| 438 | **29633** | **11** | 10 |
| 439 | **7865** | **12** | 9 |
| 440 | **7906** | **12** | 10 |
| 441 | **7972** | **12** | 10 |
| 442 | **8015** | **12** | 10 |
| 443 | **11052** | **12** | 10 |
| 444 | **11087** | **12** | 9 |
| 445 | **11098** | **12** | 9 |
| 446 | **11100** | **12** | 9 |
| 447 | **11108** | **12** | 11 |
| 448 | **11113** | **12** | 10 |
| 449 | **11123** | **12** | 10 |
| 450 | **11128** | **12** | 10 |
| 451 | **11138** | **12** | 10 |
| 452 | **27784** | **12** | 9 |
| 453 | **27848** | **12** | 9 |
| 454 | **28078** | **12** | 10 |
| 455 | **28737** | **12** | 9 |
| 456 | **28766** | **12** | 9 |
| 457 | **29064** | **12** | 10 |
| 458 | **29088** | **12** | 9 |
| 459 | **29186** | **12** | 9 |
| 460 | **6180** | **13** | 10 |
| 461 | **7878** | **13** | 10 |
| 462 | **11121** | **13** | 10 |
| 463 | **27754** | **13** | 10 |
| 464 | **28948** | **13** | 9 |
| 465 | **29406** | **13** | 10 |
| 466 | **29538** | **13** | 10 |
| 467 | **29582** | **13** | 10 |
| 468 | **28131** | **14** | 10 |
| 469 | **29556** | **14** | 10 |
| 470 | **28403** | **15** | 10 |
| 471 | **28597** | **15** | 10 |
| 472 | **29486** | **15** | 10 |
| 473 | **28544** | **16** | 10 |
| 474 | **29393** | **16** | 9 |
| 475 | **28727** | **17** | 9 |
| 476 | **7932** | **ND^(1)^** | 10 |
| 477 | **10761** | **ND** | 9 |
| 478 | **10792** | **No RD2^(2)^** | - |
| 479 | **10815** | **ND** | 9 |
| 480 | **27813** | **ND** | 9 |
| 481 | **28259** | **ND** | 11 |
| 482 | **28363** | **ND** | 9 |
| 483 | **28575** | **No RD2** | - |
| 484 | **28916** | **ND** | 9 |
| 485 | **28928** | **ND** | 11 |
| 486 | **29127** | **ND** | 9 |
| 487 | **29277** | **ND** | 10 |
| 488 | **29336** | **ND** | 9 |
| 489 | **29357** | **ND** | 11 |
| 490 | **29522** | **ND** | 9 |
| 491 | **29525** | **ND** | 9 |
| 492 | **29536** | **ND** | 10 |
| 493 | **29548** | **ND** | 9 |

^(1)^ Not determined

**^(2)^** The RD2 ICE element is not present
