# Supplementary material for: Genetic heterogeneity of the Spy1336/R28—Spy1337 virulence axis in Streptococcus pyogenes and effect on gene transcript levels and pathogenesis
Source: PLoS One. 2020 Mar 26;15(3):e0229064. doi: 10.1371/journal.pone.0229064 (PMC7098570; doi:10.1371/journal.pone.0229064)
Supplement: S3 Table — (DOCX) [file pone.0229064.s007.docx]

**S3 Table. HT*_Spy1336-7_* alleles found in 2,074 *emm28* GAS invasive strains**

| **Allele^(1)^** | **DNA Sequence^(2)^** | **HT length** | **Count^(3)^** |
| --- | --- | --- | --- |
| 1^(4)^ | TTTTATCTAATCTAATCTGC**TTTTTTTTTT** ATATATAATTTGACTTTTTC | 10 | **1226** |
| 2 | TTTTATCTAATCTAATCTGC**TTTTTTTTT** ATATATAATTTGACTTTTTC | 9 | **650** |
| 3 | TTTTATCTAATCTAATCTGC**TTTTTTTTTTT** ATATATAATTTGACTTTTTC | 11 | **128** |
| 4 | TTTTATCTAATCTAATCTGC**TTTTTTTTAT** ATATATAATTTGACTTTTTC | 8 | **15** |
| 5 | TTTTATCTAATCTAATCTGC**TTTTTTTTTTTT** ATATATAATTTGACTTTTTC | 12 | **11** |
| 6 | TTTTATCTAATCTAATCTGC**TTTTTTTTTAT** ATATATAATTTGACTTTTTC | 9 | **7** |
| 7 | TTTTATCTAATCTAATCTGC**TTTTTTTATAT** ATATATAATTTGACTTTTTC | 7 | **5** |
| 8 | TTTTATCTAATCTAATCTGC**TTTTTTTT** ATATATAATTTGACTTTTTC | 8 | **4** |
| 9 | TTTTATCTAATCTAATCTGC**TTTTTTTTTT** ATAT**G**TAATTTGACTTTTTC | 10 | **4** |
| 10 | **----**ATCTAATCTAATCTGC**TTTTTTTTTT** ATATATAATTTGACTTTTTC | 10 | **2** |
| 11 | TTTTATCTAATCTAATCTGC**TTTTTTTTT** ATATATAATTTGACTTTTT**-** | 9 | **2** |
| 12 | TTTTATCTAATCTAATCTGC**TTTTTTTTTT** ATATATAATTTGACTTTTT**-** | 10 | **2** |
| 13 | **---------**ATCTAATCTGC**TTTTTTTTTT** ATATATAATTTGACTTTTTC | 10 | **1** |
| 14 | **-------**TAATCTAATCTTC**TTTTTTTTTT** ATATATAATTTGACTTTTTC | 10 | **1** |
| 15 | **---**TATCTAATCTAATCTGC**TTTTTTTTT** ATATATAATTTGACTTTTTC | 9 | **1** |
| 16 | **---**TATCTAATCTAATCTGC**TTTTTTTTTT** ATATATAATTTGACTTTTTC | 10 | **1** |
| 17 | TTTTATCTAAT**A**TAATCTGC**TTTTTTTTTT** ATATATAATTTGACTTTTTC | 10 | **1** |
| 18 | TTTTATCTAATC**A**AATCTGC**TTTTTTTTT** ATATATAATTTGACTTTTTC | 9 | **1** |
| 19 | TTTTATCTAATCTAATCTGC**CTTTTTTTTT** ATATATAATTTGACTTTTTC | 9 | **1** |
| 20 | TTTTATCTAATCTAATCTGC**TTTTTTATAT** ATATATAATTTGACTTTTTC | 6 | **1** |
| 21 | TTTTATCTAATCTAATCTGC**TTTTTTTAT**  ATATATAATTTGACTTTTTC | 7 | **1** |
| 22 | TTTTATCTAATCTAATCTGC**TTTTTTTATT** ATATATAATTTGACTTTTTC | 7 | **1** |
| 23 | TTTTATCTAATCTAATCTGC**TTTTTTTT** ATATATAATTTGACTTTTT**-** | 8 | **1** |
| 24 | TTTTATCTAATCTAATCTGC**TTTTTTTTT** ATAT**G**TAATTTGACTTTTTC | 9 | **1** |
| 25 | TTTTATCTAATCTAATCTGC**TTTTTTTTTT** ATAT**T**TAATTTGACTTTTTC | 10 | **1** |
| 26 | TTTTATCTAATCTAATCTGC**TTTTTTTTTT** AT**T**TATAATTTGACTTTTTC | 10 | **1** |
| 27 | TTTTATCTAATCTAATCTGC**TTTTTTTTTTT** **--**ATATAATTTGACTTTTTC | 11 | **1** |
| 28 | TTTTATCTAATCTAATCTGC**TTTTTTTTTTT** ATAT**G**TAATTTGACTTTTTC | 11 | **1** |
| 29 | TTTTATCTAATCTAATCTGC**TTTTTTTTTTTTT** ATATATAATTTGACTTTTTC | 13 | **1** |
| 30 | TTTTATCTAATCTA**T**TCTGC**TTTTTTTTTT** ATATATAATTTGACTTTTTC | 10 | **1** |
| Total |  |  | **2074** |

**^(1)^ Allele number**

**^(2)^ DNA sequence comprising 20 nucleotides upstream and downstream from HT*_Spy1336-7_***

**^(3)^ Count refers to the number of strains found to contain a specific allele.**

**^(4)^ Alleles in grey contain indels in HT*_Spy1336-7_* exclusively, and are shown in Figure 2B.**

**SNPs and additional indels are indicated in red. Deletions are indicated by -**
