# Supplementary material for: Genetic heterogeneity of the Spy1336/R28—Spy1337 virulence axis in Streptococcus pyogenes and effect on gene transcript levels and pathogenesis
Source: PLoS One. 2020 Mar 26;15(3):e0229064. doi: 10.1371/journal.pone.0229064 (PMC7098570; doi:10.1371/journal.pone.0229064)
Supplement: S4 Table — (DOCX) [file pone.0229064.s008.docx]

**S4 Table. Differentially expressed genes comparing MGAS27961Δ*Spy1337* to the isogenic parental strain MGAS27961-10T**

**A. Upregulated genes.**

|  | **Growth^(1)^** | **Spy** | **gene** | **FC^(2)^** | **Function** | | | | |
| --- | --- | --- | --- | --- | --- | --- | --- | --- | --- |
| 1 | ME**^(3)^** | Spy0023 | *purL* | 2.2 | Phosphoribosylformylglycinamidine synthase | | |  |  |
| 2 | ME | Spy0169 | *-* | 1.7 | Transposase |  |  |  |  |
| 3 | ME | Spy0170 | *-* | 1.9 | Transposase |  |  |  |  |
| 4 | ME | Spy0184 | *rivR* | 1.7 | RofA-related transcriptional regulator | | |  |  |
| 5 | ME | Spy0478 | *-* | 2.1 | Thiamine transporter | |  |  |  |
| 6 | ME | Spy0873 | *-* | 1.5 | Mg2+/citrate complex secondary transporter | | |  |  |
| 7 | ME | Spy0878 | *citD* | 1.5 | Citrate lyase acyl carrier protein | |  |  |  |
| 8 | ME | Spy0899 | *fhs.1* | 1.8 | Formate--tetrahydrofolate ligase | |  |  |  |
| 9 | ME | Spy1209 | *-* | 1.5 | Xaa-His dipeptidase | |  |  |  |
| 10 | ME | Spy1210 | *-* | 1.5 | Arginine/ornithine antiporter | |  |  |  |
| 1 | ES**^(4)^** | Spy0106 | *-* | 2.6 | Sortase |  |  |  |  |
| 2 | ES | Spy0107 | *cpa* | 1.7 | Collagen-binding protein | |  |  |  |
| 3 | ES | Spy0108 | *-* | 1.6 | Signal peptidase I | |  |  |  |
| 4 | ES | Spy0109 | *-* | 1.5 | Hypothetical protein | |  |  |  |
| 5 | ES | Spy0110 | *eftLSL.B* | 1.5 | Hypothetical exported protein | |  |  |  |
| 6 | ES | Spy0111 | *-* | 1.8 | Hypothetical protein | |  |  |  |
| 7 | ES | Spy0112 | *-* | 2.5 | Transcriptional regulator, AraC family | | |  |  |
| 8 | ES | Spy0117 | *atoB* | 2.1 | Acetyl-CoA acetyltransferase | |  |  |  |
| 9 | ES | Spy0118 | *atoD.2* | 2.0 | Acetate CoA-transferase alpha subunit | | |  |  |
| 10 | ES | Spy0119 | *atoD.1* | 2.0 | Acetyl-CoA:acetoacetyl-CoA transferase beta subunit | | | |  |
| 11 | ES | Spy0121 | *ridA* | 3.3 | RidA-enamine deaminase-family protein | | |  |  |
| 12 | ES | Spy0122 | *sloR* | 4.3 | Transcriptional regulator | |  |  |  |
| 13 | ES | Spy0123 | *-* | 4.3 | Hypothetical protein | |  |  |  |
| 14 | ES | Spy0124 | *ntpI* | 4.5 | V-type sodium ATP synthase subunit I | | |  |  |
| 15 | ES | Spy0125 | *ntpK* | 4.7 | V-type sodium ATP synthase subunit K | | |  |  |
| 16 | ES | Spy0126 | *ntpE* | 5.0 | V-type sodium ATP synthase subunit E | | |  |  |
| 17 | ES | Spy0127 | *ntpC* | 4.9 | V-type ATP synthase subunit C | |  |  |  |
| 18 | ES | Spy0128 | *ntpF* | 5.2 | V-type sodium ATP synthase subunit F | | |  |  |
| 19 | ES | Spy0129 | *ntpA* | 4.4 | V-type sodium ATP synthase subunit A | | |  |  |
| 20 | ES | Spy0130 | *ntpB* | 4.0 | V-type sodium ATP synthase subunit B | | |  |  |
| 21 | ES | Spy0131 | *ntpD* | 3.9 | V-type sodium ATP synthase subunit D | | |  |  |
| 22 | ES | Spy0134 | *purA* | 1.7 | Adenylosuccinate synthetase | |  |  |  |
| 23 | ES | Spy0135 | *-* | 1.7 | Nucleoside-binding protein | |  |  |  |
| 24 | ES | Spy0157 | *polA* | 1.7 | DNA polymerase I | |  |  |  |
| 25 | ES | Spy0184 | *rivR* | 1.6 | RofA-related transcriptional regulator | | |  |  |
| 26 | ES | Spy0210 | *-* | 3.2 | Hypothetical membrane spanning protein | | |  |  |
| 27 | ES | Spy0211 | *nanH* | 3.4 | N-acetylneuraminate lyase | |  |  |  |
| 28 | ES | Spy0212 | *-* | 3.7 | N-acetylmannosamine kinase | |  |  |  |
| 29 | ES | Spy0213 | *-* | 2.0 | Transcriptional regulator, RpiR family | | |  |  |
| 30 | ES | Spy0287 | *-* | 1.6 | Acylphosphatase | |  |  |  |
| 31 | ES | Spy0329 | *prtS* | 2.6 | Lactocepin |  |  |  |  |
| 32 | ES | Spy0335 | *nrdI* | 1.7 | Hypothetical protein | |  |  |  |
| 33 | ES | Spy0336 | *nrdE.1* | 1.7 | Ribonucleoside-diphosphate reductase alpha chain | | | |  |
| 34 | ES | Spy0338 | *-* | 1.7 | Hypothetical protein | |  |  |  |
| 35 | ES | Spy0361 | *ftsK* | 1.5 | Cell division protein | |  |  |  |
| 36 | ES | Spy0398 | *-* | 1.6 | ATPase |  |  |  |  |
| 37 | ES | Spy0409 | *gloA* | 1.5 | Lactoylglutathione lyase | |  |  |  |
| 38 | ES | Spy0410 | *-* | 1.5 | NAD(P)H-dependent quinone reductase | | |  |  |
| 39 | ES | Spy0432 | *-* | 1.5 | Hypothetical protein | |  |  |  |
| 40 | ES | Spy0433 | *metK1* | 1.6 | S-adenosylmethionine synthetase | |  |  |  |
| 41 | ES | Spy0434 | *-* | 1.7 | Hypothetical membrane associated protein | | |  |  |
| 42 | ES | Spy0435 | *-* | 1.6 | Involved in cell wall biogenesis | |  |  |  |
| 43 | ES | Spy0436 | *-* | 1.6 | Hypothetical protein | |  |  |  |
| 44 | ES | Spy0498 | *agaD* | 3.3 | PTS system, N-acetylgalactosamine-specific IID component | | | | |
| 45 | ES | Spy0499 | *agaC* | 3.5 | PTS system, N-acetylgalactosamine-specific IIC component | | | | |
| 46 | ES | Spy0500 | *agaV* | 3.6 | PTS system, N-acetylgalactosamine-specific IIB component | | | | |
| 47 | ES | Spy0501 | *ugl* | 3.3 | Unsaturated glucuronyl hydrolase | |  |  |  |
| 48 | ES | Spy0502 | *agaF* | 2.9 | PTS system, N-acetylgalactosamine-specific IIA component | | | | |
| 49 | ES | Spy0503 | *idnO* | 1.8 | Gluconate 5-dehydrogenase | |  |  |  |
| 50 | ES | Spy0504 | *-* | 1.5 | Galactose-6-phosphate isomerase LacB subunit | | | |  |
| 51 | ES | Spy0506 | *kgdA* | 1.5 | 2-dehydro-3-deoxyphosphogluconate aldolase | | | |  |
| 52 | ES | Spy0536 | *-* | 1.8 | Transposase |  |  |  |  |
| 53 | ES | Spy0537 | *-* | 1.9 | Transposase |  |  |  |  |
| 54 | ES | Spy0538 | *-* | 1.8 | Transcriptional regulator | |  |  |  |
| 55 | ES | Spy0540 | *sagA* | 1.8 | Streptolysin S precursor | |  |  |  |
| 56 | ES | Spy0542 | *sagC* | 1.6 | Streptolysin S biosynthesis protein | | |  |  |
| 57 | ES | Spy0543 | *sagD* | 1.7 | Streptolysin S biosynthesis protein | | |  |  |
| 58 | ES | Spy0544 | *sagE* | 1.8 | Streptolysin S putative self-immunity protein | | |  |  |
| 59 | ES | Spy0545 | *sagF* | 1.8 | Streptolysin S biosynthesis protein | | |  |  |
| 60 | ES | Spy0546 | *sagG* | 1.9 | Streptolysin S export ATP-binding protein | | |  |  |
| 61 | ES | Spy0547 | *sagH* | 1.8 | Streptolysin S export transmembrane protein | | |  |  |
| 62 | ES | Spy0548 | *sagI* | 1.7 | Streptolysin S export transmembrane protein | | |  |  |
| 63 | ES | Spy0549 | *-* | 1.5 | Endonuclease/exonuclease/phosphatase family protein | | | | |
| 64 | ES | Spy0577 | *mscL* | 2.2 | Large-conductance mechanosensitive channel | | |  |  |
| 65 | ES | Spy0593 | *pepT* | 1.5 | Peptidase T |  |  |  |  |
| 66 | ES | Spy0594 | *ebsA* | 1.7 | Pore forming protein | |  |  |  |
| 67 | ES | Spy0595 | *-* | 1.8 | Ferredoxin |  |  |  |  |
| 68 | ES | Spy0596 | *-* | 2.0 | Hypothetical membrane associated protein | | |  |  |
| 69 | ES | Spy0597 | *cmk* | 1.7 | Cytidylate kinase |  |  |  |  |
| 70 | ES | Spy0612 | *capA* | 1.8 | Capsule biosynthesis protein | |  |  |  |
| 71 | ES | Spy0661 | *-* | 1.5 | Two component system histidine kinase | | |  |  |
| 72 | ES | Spy0674 | *clpL* | 3.8 | ATP-dependent protease ATP-binding subunit | | |  |  |
| 73 | ES | Spy0731 | *acoB* | 1.5 | Pyruvate dehydrogenase E1 component beta subunit | | | |  |
| 74 | ES | Spy0732 | *acoC* | 1.6 | Dihydrolipoamide acetyltransferase component of pyruvate DH | | | | |
| 75 | ES | Spy0733 | *-* | 1.6 | Hypothetical protein | |  |  |  |
| 76 | ES | Spy0734 | *acoL* | 1.6 | Dihydrolipoamide dehydrogenase | |  |  |  |
| 77 | ES | Spy0735 | *-* | 1.6 | Hypothetical protein | |  |  |  |
| 78 | ES | Spy0885 | *xerD* | 1.6 | Recombinase |  |  |  |  |
| 79 | ES | Spy0894 | *pdxK* | 1.9 | Hypothetical membrane spanning protein | | |  |  |
| 80 | ES | Spy0895 | *-* | 2.0 | Pyridoxine kinase | |  |  |  |
| 81 | ES | Spy0896 | *-* | 2.2 | Transcriptional regulator, GntR family | | |  |  |
| 82 | ES | Spy0943 | *-* | 2.2 | General stress protein, Gls24 family | | |  |  |
| 83 | ES | Spy0944 | *-* | 2.3 | Hypothetical protein | |  |  |  |
| 84 | ES | Spy0945 | *-* | 2.3 | General stress protein, Gls24 family | | |  |  |
| 85 | ES | Spy0946 | *-* | 2.4 | Hypothetical protein | |  |  |  |
| 86 | ES | Spy0947 | *-* | 2.3 | Hypothetical protein | |  |  |  |
| 87 | ES | Spy0948 | *-* | 2.0 | Integral membrane protein | |  |  |  |
| 88 | ES | Spy0958 | *glmS* | 1.5 | Isomerizing glucosamine-fructose-6-P aminotransferase | | | | |
| 89 | ES | Spy0963 | *-* | 1.7 | Transcriptional regulator, GntR family | | |  |  |
| 90 | ES | Spy0964 | *-* | 1.9 | ABC transporter ATP-binding protein | | |  |  |
| 91 | ES | Spy0965 | *-* | 1.9 | ABC transporter permease protein | |  |  |  |
| 92 | ES | Spy0999 | *-* | 2.0 | 6180.1 phage protein | |  |  |  |
| 93 | ES | Spy1009 | *-* | 1.8 | 6180.1 phage protein | |  |  |  |
| 94 | ES | Spy1012 | *-* | 1.8 | 6180.1 phage protein | |  |  |  |
| 95 | ES | Spy1014 | *-* | 1.7 | 6180.1 phage protein | |  |  |  |
| 96 | ES | Spy1015 | *-* | 2.0 | 6180.1 phage protein | |  |  |  |
| 97 | ES | Spy1017 | *-* | 2.0 | 6180.1 phage protein | |  |  |  |
| 98 | ES | Spy1018 | *-* | 1.9 | 6180.1 phage protein | |  |  |  |
| 99 | ES | Spy1019 | *-* | 2.3 | 6180.1 phage protein | |  |  |  |
| 100 | ES | Spy1020 | *-* | 2.2 | 6180.1 phage protein | |  |  |  |
| 101 | ES | Spy1036 | *glgP* | 1.6 | Glycogen phosphorylase | |  |  |  |
| 102 | ES | Spy1039 | *malE* | 1.9 | Maltose/maltodextrin-binding protein (ABC transporter) | | |  |  |
| 103 | ES | Spy1040 | *malF* | 1.5 | Maltose transport system permease protein (ABC transporter) | | |  |  |
| 104 | ES | Spy1041 | *malG* | 1.8 | Maltose transport system permease protein (ABC transporter) | | |  |  |
| 105 | ES | Spy1043 | *malA* | 2.7 | Maltodextrose utilization protein | |  |  |  |
| 106 | ES | Spy1044 | *malD* | 2.9 | Maltodextrin transport system permease protein | | | |  |
| 107 | ES | Spy1045 | *malC* | 2.9 | Maltose transport system permease protein | | |  |  |
| 108 | ES | Spy1060 | *celB* | 1.6 | PTS system, cellobiose-specific IIC component | | |  |  |
| 109 | ES | Spy1061 | *-* | 1.8 | Hypothetical protein | |  |  |  |
| 110 | ES | Spy1062 | *celC* | 1.7 | PTS system, cellobiose-specific IIA component | | |  |  |
| 111 | ES | Spy1063 | *celA* | 1.7 | PTS system, cellobiose-specific IIB component | | |  |  |
| 112 | ES | Spy1064 | *-* | 1.6 | Transcription antiterminator, BglG family | | |  |  |
| 113 | ES | Spy1065 | *-* | 2.0 | Outer surface protein | |  |  |  |
| 114 | ES | Spy1066 | *bglA.2* | 1.9 | Beta-glucosidase | |  |  |  |
| 115 | ES | Spy1085 | *-* | 1.6 | RD.1 transcriptional regulator, MarR family | | |  |  |
| 116 | ES | Spy1123 | *-* | 1.6 | CPBP family intramembrane metalloprotease | | |  |  |
| 117 | ES | Spy1161 | *-* | 1.5 | Lead, cadmium, zinc and mercury transporting ATPase | | | | |
| 118 | ES | Spy1179 | *clpE* | 1.7 | ATP-dependent Clp protease ATP-binding subunit | | | |  |
| 119 | ES | Spy1208 | *arcC* | 2.3 | Carbamate kinase | |  |  |  |
| 120 | ES | Spy1209 | *-* | 2.3 | Xaa-His dipeptidase | |  |  |  |
| 121 | ES | Spy1218 | *-* | 1.5 | Two-component sensor kinase | |  |  |  |
| 122 | ES | Spy1289 | *-* | 1.9 | Hypothetical cytosolic protein | |  |  |  |
| 123 | ES | Spy1290 | *-* | 2.0 | Hypothetical protein | |  |  |  |
| 124 | ES | Spy1291 | *-* | 2.0 | Hypothetical protein | |  |  |  |
| 125 | ES | Spy1292 | *-* | 1.8 | Hypothetical cytosolic protein | |  |  |  |
| 126 | ES | Spy1293 | *-* | 1.6 | Hypothetical protein | |  |  |  |
| 127 | ES | Spy1294 | *-* | 1.6 | Hypothetical protein | |  |  |  |
| 128 | ES | Spy1295 | *-* | 1.6 | ATP-dependent RNA helicase | |  |  |  |
| 129 | ES | Spy1307 | *-* | 2.0 | RD.2 hypothetical exported protein | | |  |  |
| 130 | ES | Spy1308 | *-* | 2.0 | RD.2 hypothetical exported protein | | |  |  |
| 131 | ES | Spy1311 | *-* | 1.6 | RD.2 DNA segregation ATPase related protein | | |  |  |
| 132 | ES | Spy1314 | *-* | 1.7 | RD.2 hypothetical protein | |  |  |  |
| 133 | ES | Spy1322 | *-* | 1.7 | RD.2 FtsK/SpoIIIE family | |  |  |  |
| 134 | ES | Spy1323 | *-* | 1.8 | RD.2 hypothetical protein | |  |  |  |
| 135 | ES | Spy1324 | *-* | 1.6 | RD.2 hypothetical protein | |  |  |  |
| 136 | ES | Spy1325 | *-* | 1.7 | RD.2 putative cell surface protein | |  |  |  |
| 137 | ES | Spy1349 | *-* | 3.1 | Sugar-binding protein | |  |  |  |
| 138 | ES | Spy1350 | *-* | 3.1 | Sugar transport system permease protein | | |  |  |
| 139 | ES | Spy1351 | *-* | 2.9 | Sugar transport system permease protein | | |  |  |
| 140 | ES | Spy1352 | *nagC* | 3.3 | Glucokinase or transcriptional regulator |  |  |  |  |
| 141 | ES | Spy1353 | *-* | 3.7 | Hypothetical protein | |  |  |  |
| 142 | ES | Spy1354 | *-* | 3.3 | Beta-glucosidase | |  |  |  |
| 143 | ES | Spy1355 | *hyl* | 3.5 | Hyaluronoglucosaminidase | |  |  |  |
| 144 | ES | Spy1356 | *-* | 3.2 | Transcriptional regulator, GntR family | | |  |  |
| 145 | ES | Spy1357 | *-* | 3.8 | Hypothetical protein | |  |  |  |
| 146 | ES | Spy1358 | *-* | 4.1 | Alpha-mannosidase | |  |  |  |
| 147 | ES | Spy1368 | *comFA* | 1.9 | ComF operon protein 1 | |  |  |  |
| 148 | ES | Spy1438 | *lacD.1* | 2.8 | Tagatose-bisphosphate aldolase | |  |  |  |
| 149 | ES | Spy1439 | *lacC.1* | 2.7 | Tagatose-6-phosphate kinase | |  |  |  |
| 150 | ES | Spy1440 | *lacB.1* | 2.5 | Galactose-6-phosphate isomerase LacB subunit | | | |  |
| 151 | ES | Spy1441 | *lacA.1* | 2.5 | Galactose-6-phosphate isomerase LacA subunit | | | |  |
| 152 | ES | Spy1447 | *copZ* | 3.1 | Copper chaperone | |  |  |  |
| 153 | ES | Spy1448 | *copA* | 3.5 | Copper-exporting ATPase | |  |  |  |
| 154 | ES | Spy1449 | *copY* | 2.3 | CopAB ATPases metal-fist type repressor | | |  |  |
| 155 | ES | Spy1486 | *dnaJ* | 3.2 | Chaperone protein | |  |  |  |
| 156 | ES | Spy1487 | *dnaK* | 3.3 | Chaperone protein | |  |  |  |
| 157 | ES | Spy1488 | *grpE* | 3.1 | Hypothetical protein | |  |  |  |
| 158 | ES | Spy1489 | *hrcA* | 2.4 | Heat-inducible transcription repressor | | |  |  |
| 159 | ES | Spy1511 | *cbiO* | 1.5 | Cobalt transport ATP-binding protein CbiO | | |  |  |
| 160 | ES | Spy1512 | *cbiQ* | 1.5 | Cobalt transport protein cbiQ | |  |  |  |
| 161 | ES | Spy1514 | *-* | 1.6 | ABC transporter ATP-binding protein | | |  |  |
| 162 | ES | Spy1515 | *-* | 1.8 | ABC transporter ATP-binding protein | | |  |  |
| 163 | ES | Spy1516 | *fhuC* | 1.7 | Ferrichrome transport ATP-binding protein | | |  |  |
| 164 | ES | Spy1517 | *fhuB* | 1.6 | Ferrichrome transport system permease protein | | | |  |
| 165 | ES | Spy1518 | *fhuD* | 1.5 | Ferrichrome-binding protein | |  |  |  |
| 166 | ES | Spy1527 | *endoS* | 2.7 | Endo-beta-N-acetylglucosaminidase F2 precursor | | | |  |
| 167 | ES | Spy1528 | *-* | 2.3 | Hypothetical protein | |  |  |  |
| 168 | ES | Spy1529 | *scrA* | 2.5 | PTS system, sucrose-specific IIABC component | | | |  |
| 169 | ES | Spy1543 | *-* | 1.5 | Hypothetical protein | |  |  |  |
| 170 | ES | Spy1603 | *-* | 1.6 | Hypothetical protein | |  |  |  |
| 171 | ES | Spy1605 | *thiD* | 1.6 | Hydroxymethylpyrimidine kinase | |  |  |  |
| 172 | ES | Spy1606 | *-* | 1.6 | tRNA pseudouridine synthase A | |  |  |  |
| 173 | ES | Spy1619 | *salX* | 1.6 | Lantibiotic transport ATP-binding protein | | |  |  |
| 174 | ES | Spy1620 | *salB* | 1.6 | Serine (threonine) dehydratase | |  |  |  |
| 175 | ES | Spy1621 | *salA* | 2.0 | Lantibiotic salivaricin A | |  |  |  |
| 176 | ES | Spy1622 | *lacG* | 2.4 | 6-phospho-beta-galactosidase | |  |  |  |
| 177 | ES | Spy1623 | *lacE* | 3.1 | PTS system, lactose-specific IIBC component | | |  |  |
| 178 | ES | Spy1624 | *lacF* | 3.0 | PTS system, lactose-specific IIA component | | |  |  |
| 179 | ES | Spy1625 | *lacD.2* | 2.8 | Tagatose-bisphosphate aldolase | |  |  |  |
| 180 | ES | Spy1626 | *lacC.2* | 2.5 | Tagatose-6-phosphate kinase | |  |  |  |
| 181 | ES | Spy1627 | *lacB.2* | 2.4 | Galactose-6-phosphate isomerase LacB subunit | | | |  |
| 182 | ES | Spy1628 | *lacA.2* | 2.1 | Galactose-6-phosphate isomerase LacA subunit | | | |  |
| 183 | ES | Spy1649 | *-* | 1.9 | Transaldolase |  |  |  |  |
| 184 | ES | Spy1650 | *-* | 2.2 | Putative transport protein sgaT | |  |  |  |
| 185 | ES | Spy1651 | *-* | 2.4 | PTS system, IIB component | |  |  |  |
| 186 | ES | Spy1652 | *-* | 1.9 | Transcription antiterminator, BglG family | | |  |  |
| 187 | ES | Spy1667 | *-* | 2.2 | Hypothetical protein | |  |  |  |
| 188 | ES | Spy1668 | *pulA* | 2.1 | Pullulanase |  |  |  |  |
| 189 | ES | Spy1669 | *dexB* | 2.0 | Glucan 1,6-alpha-glucosidase | |  |  |  |
| 190 | ES | Spy1677 | *xthA* | 2.0 | Exodeoxyribonuclease III | |  |  |  |
| 191 | ES | Spy1678 | *-* | 2.4 | PTS system, glucose-specific IIABC component | | |  |  |
| 192 | ES | Spy1679 | *-* | 1.9 | Hypothetical cytosolic protein | |  |  |  |
| 193 | ES | Spy1680 | *prmA* | 1.9 | Ribosomal protein L11 methyltransferase | | |  |  |
| 194 | ES | Spy1681 | *-* | 1.8 | Hypothetical protein | |  |  |  |
| 195 | ES | Spy1683 | *trpG* | 1.7 | *p*-aminobenzoate synthase glutamine amidotransferase component II | | | | |
| 196 | ES | Spy1684 | *-* | 1.8 | ATPase, AAA family | |  |  |  |
| 197 | ES | Spy1686 | *flaR* | 1.7 | DNA topology modulation protein flar-related protein | | | | |
| 198 | ES | Spy1699 | *-* | 1.5 | Cell surface protein | |  |  |  |
| 199 | ES | Spy1706 | *-* | 2.8 | Hypothetical protein | |  |  |  |
| 200 | ES | Spy1707 | *isp* | 3.1 | Immunogenic secreted protein | |  |  |  |
| 201 | ES | Spy1708 | *ihk* | 2.9 | Two component system histidine kinase | | |  |  |
| 202 | ES | Spy1709 | *irr* | 2.9 | Two-component response regulator | | |  |  |
| 203 | ES | Spy1710 | *-* | 3.5 | ABC transporter permease protein | |  |  |  |
| 204 | ES | Spy1711 | *-* | 4.8 | ABC transporter ATP-binding protein | | |  |  |
| 205 | ES | Spy1712 | *-* | 4.6 | Component of efflux system | |  |  |  |
| 206 | ES | Spy1713 | *-* | 3.7 | Hypothetical protein | |  |  |  |
| 207 | ES | Spy1718 | *-* | 2.6 | Protein export protein prsA precursor | | |  |  |
| 208 | ES | Spy1719 | *-* | 2.7 | Hypothetical protein | |  |  |  |
| 209 | ES | Spy1720 | *-* | 2.6 | Streptopain fragment | |  |  |  |
| 210 | ES | Spy1721 | *speB* | 2.7 | Streptococcal pyrogenic exotoxin B | | |  |  |
| 211 | ES | Spy1725 | *mf* | 2.1 | Mitogenic factor |  |  |  |  |
| 212 | ES | Spy1726 | *-* | 2.3 | Hypothetical protein | |  |  |  |
| 213 | ES | Spy1734 | *-* | 1.7 | Sorbitol operon regulator | |  |  |  |
| 214 | ES | Spy1747 | *groEL* | 2.3 | 60 kDa chaperonin | |  |  |  |
| 215 | ES | Spy1759 | *-* | 2.2 | Hypothetical cytosolic protein | |  |  |  |
| 216 | ES | Spy1760 | *-* | 2.3 | Amino acid permease | |  |  |  |
| 217 | ES | Spy1761 | *hutH* | 2.2 | Histidine ammonia-lyase | |  |  |  |
| 218 | ES | Spy1773 | *nrdG* | 2.5 | Ribonucleoside-triphosphate reductase activating protein | | | | |
| 219 | ES | Spy1774 | *-* | 2.6 | Acetyltransferase | |  |  |  |
| 220 | ES | Spy1775 | *-* | 2.7 | Putative oxidoreductase | |  |  |  |
| 221 | ES | Spy1776 | *-* | 3.0 | Hypothetical protein | |  |  |  |
| 222 | ES | Spy1777 | *nrdD* | 3.6 | Anaerobic ribonucleoside-triphosphate reductase | | | |  |
| 223 | ES | Spy1805 | *-* | 1.9 | 6180.3 phage protein | |  |  |  |
| 224 | ES | Spy1847 | *-* | 2.3 | 6180.4 phage protein | |  |  |  |
| 225 | ES | Spy1873 | *sdhB* | 3.4 | L-serine dehydratase | |  |  |  |
| 226 | ES | Spy1874 | *sdhA* | 3.7 | L-serine dehydratase | |  |  |  |

**B. Downregulated genes.**

|  | **Growth** | **Spy** | **gene** | **FC** | **Function** | | |
| --- | --- | --- | --- | --- | --- | --- | --- |
| 1 | ME | Spy0339 | *-* | -2.3 | Transposase |  |  |
| 2 | ME | Spy0540 | *sagA* | -2.7 | Streptolysin S precursor | |  |
| 3 | ME | Spy0541 | *sagB* | -2.1 | Streptolysin S biosynthesis protein | | |
| 4 | ME | Spy0542 | *sagC* | -1.7 | Streptolysin S biosynthesis protein | | |
| 5 | ME | Spy0543 | *sagD* | -1.7 | Streptolysin S biosynthesis protein | | |
| 6 | ME | Spy0544 | *sagE* | -1.7 | Streptolysin S putative self-immunity protein | | |
| 7 | ME | Spy0545 | *sagF* | -1.7 | Streptolysin S biosynthesis protein | | |
| 8 | ME | Spy0546 | *sagG* | -1.6 | Streptolysin S export ATP-binding protein | | |
| 9 | ME | Spy0547 | *sagH* | -1.6 | Streptolysin S export transmembrane protein | | |
| 10 | ME | Spy0548 | *sagI* | -1.6 | Streptolysin S export transmembrane protein | | |
| 11 | ME | Spy0951 | *-* | -1.5 | Hypothetical protein | |  |
| 12 | ME | Spy0999 | *-* | -1.5 | 6180.1 phage protein | |  |
| 13 | ME | Spy1001 | *-* | -1.6 | 6180.1 phage protein | |  |
| 14 | ME | Spy1336 | *Spy1336/R28* | -89.2 | RD.2 R28 virulence factor | | |
| 15 | ME | Spy1337 | *Spy1337* | -664.8 | RD.2 Spy1337 transcriptional regulator, AraC family | | |
| 16 | ME | Spy1338 | *-* | -1.6 | Phospho-2-dehydro-3-deoxyheptonate aldolase | | |
| 17 | ME | Spy1339 | *aroB* | -1.5 | 3-dehydroquinate synthase | |  |
| 18 | ME | Spy1340 | *-* | -1.8 | Hypothetical protein | |  |
| 19 | ME | Spy1482 | *acpP.2* | -1.6 | Acyl carrier protein | |  |
| 20 | ME | Spy1483 | *fabH* | -2.0 | 3-oxoacyl-[acyl-carrier-protein] synthase III | | |
| 21 | ME | Spy1484 | *fabT* | -1.6 | Transcriptional regulator, MarR family | | |
| 22 | ME | Spy1653 | *-* | -1.5 | Hypothetical protein | |  |
| 23 | ME | Spy1740 | *-* | -2.1 | Translation initiation inhibitor | |  |
| 24 | ME | Spy1799 | *-* | -1.5 | 6180.3 phage protein | |  |
| 1 | ES | Spy0014 | *-* | -2.3 | Amino acid permease | |  |
| 2 | ES | Spy0063 | *secY* | -1.7 | Protein translocase subunit | |  |
| 3 | ES | Spy0064 | *adk* | -2.2 | Adenylate kinase |  |  |
| 4 | ES | Spy0065 | *infA* | -1.7 | Translation initiation factor IF-1 | |  |
| 5 | ES | Spy0066 | *rpmJ* | -1.7 | LSU ribosomal protein L36P | |  |
| 6 | ES | Spy0067 | *rpsM* | -1.7 | SSU ribosomal protein S13P | |  |
| 7 | ES | Spy0068 | *rpsK* | -1.7 | SSU ribosomal protein S11 | |  |
| 8 | ES | Spy0069 | *rpoA* | -1.7 | RNA polymerase alpha chain | |  |
| 9 | ES | Spy0070 | *rplQ* | -1.7 | LSU ribosomal protein L17P | |  |
| 10 | ES | Spy0079 | *tyrS* | -2.6 | Tyrosyl-tRNA synthetase | |  |
| 11 | ES | Spy0092 | *ackA* | -1.6 | Acetate kinase |  |  |
| 12 | ES | Spy0100 | *ssb* | -2.3 | Single-strand DNA binding protein | | |
| 13 | ES | Spy0144 | *metB* | -1.7 | Cystathionine beta-lyase | |  |
| 14 | ES | Spy0153 | *-* | -1.5 | Transcription antiterminator, BglG family | | |
| 15 | ES | Spy0261 | *-* | -1.9 | Hypothetical cytosolic protein | |  |
| 16 | ES | Spy0262 | *-* | -1.5 | ABC transporter substrate-binding protein | | |
| 17 | ES | Spy0270 | *gidB* | -1.7 | Glucose inhibited division protein B | | |
| 18 | ES | Spy0271 | *lemA* | -1.6 | Hypothetical protein | |  |
| 19 | ES | Spy0272 | *-* | -1.5 | Heat shock protein HtpX | |  |
| 20 | ES | Spy0359 | *mtsC* | -1.6 | Manganese transport system membrane protein | | |
| 21 | ES | Spy0364 | *rplA* | -2.0 | LSU ribosomal protein L1P | |  |
| 22 | ES | Spy0405 | *pcp* | -1.9 | Pyrrolidone-carboxylate peptidase | | |
| 23 | ES | Spy0427 | *smc* | -1.6 | Chromosome partition protein smc | | |
| 24 | ES | Spy0444 | *-* | -1.6 | RelB-domain protein | |  |
| 25 | ES | Spy0445 | *-* | -1.7 | ParE-domain protein | |  |
| 26 | ES | Spy0446 | *-* | -1.7 | Hypothetical cytosolic protein | |  |
| 27 | ES | Spy0447 | *-* | -1.8 | Hypothetical protein | |  |
| 28 | ES | Spy0449 | *-* | -1.9 | Transposase |  |  |
| 29 | ES | Spy0481 | *-* | -1.5 | Hypothetical exported protein | |  |
| 30 | ES | Spy0553 | *atpE* | -1.8 | ATP synthase C chain | |  |
| 31 | ES | Spy0554 | *atpB* | -2.0 | ATP synthase A chain | |  |
| 32 | ES | Spy0555 | *atpF* | -1.9 | ATP synthase B chain | |  |
| 33 | ES | Spy0556 | *atpH* | -1.9 | ATP synthase delta chain | |  |
| 34 | ES | Spy0557 | *atpA* | -1.7 | ATP synthase alpha chain | |  |
| 35 | ES | Spy0558 | *atpG* | -1.6 | ATP synthase gamma chain | |  |
| 36 | ES | Spy0559 | *atpD* | -1.5 | ATPase, beta subunit | |  |
| 37 | ES | Spy0560 | *atpC* | -1.5 | ATP synthase epsilon chain | |  |
| 38 | ES | Spy0561 | *-* | -1.5 | Hypothetical membrane associated protein | | |
| 39 | ES | Spy0598 | *infC* | -1.9 | Bacterial protein translation initiation factor IF-3 | | |
| 40 | ES | Spy0599 | *rpl36* | -2.0 | LSU ribosomal protein L35P | |  |
| 41 | ES | Spy0600 | *rplT* | -2.1 | LSU ribosomal protein L20P | |  |
| 42 | ES | Spy0630 | *-* | -1.7 | RNA binding protein | |  |
| 43 | ES | Spy0631 | *-* | -1.9 | Hypothetical protein | |  |
| 44 | ES | Spy0666 | *-* | -1.9 | 3-hydroxy-3-methylglutaryl-coenzyme A reductase | | |
| 45 | ES | Spy0667 | *mvaS.1* | -2.0 | Hydroxymethylglutaryl-CoA synthase | | |
| 46 | ES | Spy0673 | *-* | -1.5 | Hypothetical protein | |  |
| 47 | ES | Spy0691 | *parE* | -1.5 | Topoisomerase IV subunit B | |  |
| 48 | ES | Spy0692 | *parC* | -1.6 | Topoisomerase IV subunit A | |  |
| 49 | ES | Spy0693 | *bcaT* | -2.2 | Branched-chain amino acid aminotransferase | | |
| 50 | ES | Spy0694 | *-* | -2.0 | Hypothetical cytosolic protein | |  |
| 51 | ES | Spy0824 | *-* | -1.8 | ATP-NAD kinase |  |  |
| 52 | ES | Spy0910 | *pgmA* | -1.6 | Phosphomannomutase | |  |
| 53 | ES | Spy0918 | *rpsT* | -1.9 | SSU ribosomal protein S20P | |  |
| 54 | ES | Spy1076 | *-* | -1.5 | Hypothetical cytosolic protein | |  |
| 55 | ES | Spy1087 | *-* | -1.7 | RD.1 hypothetical cytosolic protein | | |
| 56 | ES | Spy1088 | *-* | -1.7 | RD.1 hypothetical protein | |  |
| 57 | ES | Spy1089 | *-* | -1.6 | RD.1 hypothetical cytosolic protein | | |
| 58 | ES | Spy1090 | *-* | -1.6 | tRNA (uracil-5-)-methyltransferase | | |
| 59 | ES | Spy1098 | *grab* | -1.9 | Protein G-related alpha 2M-binding protein | | |
| 60 | ES | Spy1193 | *-* | -1.7 | Hypothetical membrane spanning protein | | |
| 61 | ES | Spy1336 | *Spy1336/R28* | -355.4 | RD.2 R28 virulence factor | | |
| 62 | ES | Spy1337 | *Spy1337* | -478.9 | RD.2 Spy1337 transcriptional regulator, AraC family | | |
| 63 | ES | Spy1338 | *-* | -2.5 | Phospho-2-dehydro-3-deoxyheptonate aldolase | | |
| 64 | ES | Spy1339 | *aroB* | -2.5 | 3-dehydroquinate synthase | |  |
| 65 | ES | Spy1340 | *-* | -2.5 | Hypothetical protein | |  |
| 66 | ES | Spy1371 | *-* | -1.7 | s1-type RNA-binding domain | |  |
| 67 | ES | Spy1372 | *-* | -1.6 | Peptidyl-prolyl cis-trans isomerase | | |
| 68 | ES | Spy1401 | *-* | -1.6 | Thioredoxin reductase | |  |
| 69 | ES | Spy1403 | *-* | -1.9 | Transporter |  |  |
| 70 | ES | Spy1404 | *-* | -1.7 | Amino acid ABC transporter permease protein | | |
| 71 | ES | Spy1434 | *-* | -1.8 | DegV family protein | |  |
| 72 | ES | Spy1463 | *lytR* | -1.5 | Transcriptional regulator, LytR family | | |
| 73 | ES | Spy1468 | *manL* | -1.8 | PTS system, mannose-specific IIAB component | | |
| 74 | ES | Spy1469 | *manM* | -1.5 | PTS system, mannose-specific IIC component | | |
| 75 | ES | Spy1482 | *acpP.2* | -2.4 | Acyl carrier protein | |  |
| 76 | ES | Spy1504 | *-* | -1.8 | Hydrolase (HAD superfamily) | |  |
| 77 | ES | Spy1591 | *pgk* | -1.6 | Phosphoglycerate kinase | |  |
| 78 | ES | Spy1643 | *cysS* | -1.5 | Cysteinyl-tRNA synthetase | |  |
| 79 | ES | Spy1654 | *-* | -2.0 | SSU ribosomal protein S15P | |  |
| 80 | ES | Spy1676 | *-* | -1.6 | NrdI.1 |  |  |
| 81 | ES | Spy1825 | *rpmG* | -2.0 | LSU ribosomal protein L33P | |  |
| 82 | ES | Spy1865 | *rpsD* | -2.0 | SSU ribosomal protein S4P | |  |

**^(1)^** Growth refers to the growth phase at which cells were collected

**^(2)^** FC, fold change. The FC threshold is 1.5

**^(3)^** ME, mid-exponential

**^(4)^** ES, early stationary
