# Supplementary material for: Genetic heterogeneity of the Spy1336/R28—Spy1337 virulence axis in Streptococcus pyogenes and effect on gene transcript levels and pathogenesis
Source: PLoS One. 2020 Mar 26;15(3):e0229064. doi: 10.1371/journal.pone.0229064 (PMC7098570; doi:10.1371/journal.pone.0229064)
Supplement: S5 Table — (DOCX) [file pone.0229064.s009.docx]

**S5 Table. Oligonucleotides used for isogenic mutant generation and**

**and PCR amplification of *Spy1336/R28***

| **Oligonucleotide** | | **DNA sequence** |
| --- | --- | --- |
| **JE410** | 5’-CGATCTCGTGCTCGATAAGCTGTTTCG-3’ | |
| **JE412**  **JE431** | 5’-CGCATTTACACTTTCTGGATCACTAGCC-3  5’-GTTTGTTGGAGAGAGATACATAAC-3’’ | |
| **JE432** | 5’-GACGAAAATGAAGTTGAACTAGG-3’ | |
| **JE433** | 5’-GCAGAACCTAAGACAGTTACCATC-3’ | |
| **HPN-seq** | 5’-ACTAAGACCAATTAGTACAGAAGCT-3’ | |
| **1336-1** | 5’- GTCCGGATCCTCACCTTCAAGTGAATCATTAGAAC -3’ | |
| **1336-2** | 5’- TTGAAGAGTCAATAGTTGCTGCATATCTCTCTCCAACAAACTTAATTA -3’ | |
| **1336-3** | 5’- TAATTAAGTTTGTTGGAGAGAGATATGCAGCAACTATTGACTCTTCAA -3’ | |
| **1336-4** | 5’- GTCCGGATCCTTTAGCTATTTCTTCTGTTTTAATA -3’ | |
| **1337-1** | 5’- GTCCGGATCCTAGGTTCTTTAGTAAGATACTTAA -3’ | |
| **1337-2** | 5’- AGCAAAATGAATATTTTTCGATTACTTTGATTTAATATGTTATCTATT -3’ | |
| **1337-3** | 5’- AATAGATAACATATTAAATCAAAGTAATCGAAAAATATTCATTTTGCT -3’ | |
| **1337-4** | 5’- GTCCGGATCCATGGACATGAAGTGTTTGTGGCAT -3’ | |
| **1336-1337-1** | 5’- GTCCGGATCCTCACCTTCAAGTGAATCATTAGAAC -3’ | |
| **1336-1337-2** | 5’- TGAAGGGAATATTAAGCAAAATGAGCCTATCGAGATTATTAATTTCGA -3’ | |
| **1336-1337-3** | 5’- TCGAAATTAATAATCTCGATAGGCTCATTTTGCTTAATATTCCCTTCA -3’ | |
| **1336-1337-4** | 5’- GTCCGGATCCTCATCAGTACTAGGTAACAGAGATA -3’ | |
