# Supplementary material for: Genetic heterogeneity of the Spy1336/R28—Spy1337 virulence axis in Streptococcus pyogenes and effect on gene transcript levels and pathogenesis
Source: PLoS One. 2020 Mar 26;15(3):e0229064. doi: 10.1371/journal.pone.0229064 (PMC7098570; doi:10.1371/journal.pone.0229064)
Supplement: S6 Table — (DOCX) [file pone.0229064.s010.docx]

**S6 Table. Isogenic mutants generated in this study**

| **Isogenic strains** | **Number of Ts**  **in HT*_Spy1336-7_*** | ***Spy1336***  **present** | ***Spy1337***  **present** |
| --- | --- | --- | --- |
| **MGAS27961-9T** | 9 | √ | √ |
| **MGAS27961-10T** | 10 | √ | √ |
| **MGAS27961-11T** | 11 | √ | √ |
| **MGAS27961-10T-Δ*Spy1336*** | 10 | - | √ |
| **MGAS27961-10T*-*Δ*Spy1337*** | 10 | √ | - |
| **MGAS27961-10T-Δ*Spy1336*/Δ*Spy1336*** | 10 | - | - |
